# *In vitro* selection of *Neisseria gonorrhoeae* unveils novel mutations associated with extended-spectrum cephalosporin resistance

**DOI:** 10.3389/fcimb.2022.924764

**Published:** 2022-07-27

**Authors:** Marcos André Schörner, Dany Mesa, Fernando Hartmann Barazzetti, Jéssica Motta Martins, Hanalydia de Melo Machado, Henrique Borges da Silva Grisard, Julia Kinetz Wachter, Márick Rodrigues Starick, Mara Cristina Scheffer, Jussara Kasuko Palmeiro, Maria Luiza Bazzo

**Affiliations:** ^1^ Laboratório de Biologia Molecular, Microbiologia e Sorologia, Departamento de Análises Clínicas, Universidade Federal de Santa Catarina, Florianópolis, SC, Brazil; ^2^ Instituto de Pesquisa Pelé Pequeno Príncipe, Curitiba, PR, Brazil; ^3^ Laboratório de Bioinformática, Programa de Pós-Graduação em Biotecnologia e Biociências, Departamento de Microbiologia, Imunologia e Parasitologia, Universidade Federal de Santa Catarina, Florianópolis, SC, Brazil; ^4^ Programa de Pós-Graduação em Farmácia, Departamento de Análises Clínicas, Universidade Federal de Santa Catarina, Florianópolis, SC, Brazil; ^5^ Programa de Pós-Graduação em Farmacologia, Departamento de Farmacologia, Universidade Federal de Santa Catarina, Florianópolis, SC, Brazil; ^6^ Laboratório de Imunofarmacologia e Doenças Infecciosas, Departamento de Microbiologia, Imunologia e Parasitologia, Universidade Federal de Santa Catarina, Florianópolis, SC, Brazil; ^7^ Laboratório de Microbiologia, Unidade do Laboratório de Análises Clínicas, Hospital Universitário Professor Polydoro Ernani de São Thiago, Universidade Federal de Santa Catarina, Florianópolis, SC, Brazil; ^8^ Centro de Ciências da Saúde, Departamento de Análises Clínicas, Universidade Federal de Santa Catarina, Florianópolis, SC, Brazil

**Keywords:** SNP, whole-genome sequencing, PBP2, *penA*, resistance induction, mutations

## Abstract

The emergence of *Neisseria gonorrhoeae* strains resistant to extended-spectrum cephalosporins (ESCs) is a worldwide concern because this class of antibiotics represents the last empirical treatment option for gonorrhea. The abusive use of antimicrobials may be an essential factor for the emergence of ESC resistance in *N. gonorrhoeae*. Cephalosporin resistance mechanisms have not been fully clarified. In this study, we mapped mutations in the genome of *N. gonorrhoeae* isolates after resistance induction with cefixime and explored related metabolic pathways. Six clinical isolates with different antimicrobial susceptibility profiles and genotypes and two gonococcal reference strains (WHO F and WHO Y) were induced with increasing concentrations of cefixime. Antimicrobial susceptibility testing was performed against six antimicrobial agents before and after induction. Clinical isolates were whole-genome sequenced before and after induction, whereas reference strains were sequenced after induction only. Cefixime resistance induction was completed after 138 subcultures. Several metabolic pathways were affected by resistance induction. Five isolates showed SNPs in PBP2. The isolates M111 and M128 (ST1407 with mosaic *penA*-34.001) acquired one and four novel missense mutations in PBP2, respectively. These isolates exhibited the highest minimum inhibitory concentration (MIC) for cefixime among all clinical isolates. Mutations in genes contributing to ESC resistance and in other genes were also observed. Interestingly, M107 and M110 (ST338) showed no mutations in key determinants of ESC resistance despite having a 127-fold increase in the MIC of cefixime. These findings point to the existence of different mechanisms of acquisition of ESC resistance induced by cefixime exposure. Furthermore, the results reinforce the importance of the gonococcal antimicrobial resistance surveillance program in Brazil, given the changes in treatment protocols made in 2017 and the nationwide prevalence of sequence types that can develop resistance to ESC.

## Introduction

*Neisseria gonorrhoeae* (gonococcus) is the causative agent of gonorrhea, a sexually transmitted infection with an estimated 87 million new cases per year globally (Rowley et al., 2019). Since the introduction of the first antimicrobial treatment for gonorrhea, gonococcus has developed antimicrobial resistance (AMR) against all classes of antimicrobials. Resistance to extended-spectrum cephalosporins (ESCs) is a current challenge worldwide, as this class of antimicrobials represents the last empirical monotherapy for gonorrhea (Unemo, 2015).

After the first report of ESC-resistant *N. gonorrhoeae* (Ameyama et al., 2002), new cases were reported in 2011 in France (Unemo et al., 2012) and in 2012 in Spain (Cámara et al., 2012), with both isolates belonging to the ST1407. This clone was responsible for a large proportion of cases of decreased susceptibility and resistance to ESCs in several countries (Unemo and Nicholas, 2012). In *N. gonorrhoeae*, resistance to cephalosporins is primarily due to mutations in penicillin-binding protein 2 (PBP2), encoded by *penA*. Some isolates have acquired several mutations in *penA* by DNA transformation and recombination with *penA* genes from commensal *Neisseria* spp. (Ameyama et al., 2002; Ito et al., 2005; Osaka et al., 2008). Besides that, mutations in porin PorB1b and in the efflux pump MtrCDE can contribute to AMR by decreasing the influx and increasing the efflux of ESCs (Hagman and Shafer, 1995; Gill et al., 1998; Olesky et al., 2002; Zhao et al., 2009; Ohneck et al., 2011; Golparian et al., 2014).

Surveillance of gonococcal antimicrobial susceptibility has stimulated changes in the treatment of gonorrhea to slow up the emergence of resistant isolates. Several reports of oral cefixime treatment failures and an increase in resistance levels raised concerns about the use of cefixime, mainly as monotherapy (Chisholm et al., 2010; Unemo and Nicholas, 2012; Allen et al., 2013; Lewis, 2015; Unemo, 2015; Barbee et al., 2018; Unemo et al., 2019). Therefore, several countries have introduced dual antimicrobial therapy, such as ceftriaxone plus azithromycin, with the possibility of replacing ceftriaxone for cefixime when administration of an intramuscular injection of ceftriaxone is contraindicated or refused (Unemo et al., 2020). In Brazil, clinical protocols for the management of sexually transmitted infections were introduced in 1993 (Moherdaui et al., 1998). Until 2017, ciprofloxacin and azithromycin were the first-line treatment for uncomplicated gonococcal urethritis (Brasil, 2015). After that, however, ceftriaxone plus azithromycin was recommended based on the findings of the first national surveillance of antimicrobial susceptibility in *N. gonorrhoeae* (Bazzo et al., 2018). Cefixime is not commercialized in Brazil, which decreases the selection pressure on *N. gonorrhoeae* isolates (Brasil, 2020).

This study aimed to evaluate the behavior of the most prevalent sequence types (STs) of *N. gonorrhoeae* isolates in Brazil upon exposure to the antimicrobial cefixime. Gonococcus isolates with borderline resistance to cefixime were identified in Brazil in 2015–2016 (Bazzo et al., 2018) and, in 2017, the country issued its first nationwide recommendation for the use of an ESC for gonococcal infection. Given that cephalosporin resistance mechanisms are not fully clarified, this study may contribute to further understanding of the molecular mechanisms of ESC resistance in *N. gonorrhoeae* driven by selective pressure.

## Materials and methods

### Bacterial isolates

Eight gonococcal isolates were selected: a single blood culture isolate from an inpatient at the University Hospital of the Federal University of Santa Catarina; five male urethral discharge isolates selected from a nationwide study, the Brazilian-GASP (Gonococcal Antimicrobial Surveillance Programme) (Bazzo et al., 2018); and two reference strains, WHO F and WHO Y (Unemo et al., 2016).

These isolates were selected based on cefixime susceptibility profile and ST. We included cefixime-susceptible isolates with an 8- to 32-fold minimum inhibitory concentration (MIC) lower than 0.125 mg/L and an MIC of 0.125 mg/L (susceptibility threshold breakpoint). ST1407 was included because of previous reports related to ESC resistance spread (Cámara et al., 2012; Unemo et al., 2012). ST338 was included because it was found to be highly prevalent in Florianópolis, Santa Catarina, Southern Brazil (Golfetto, 2018) (**Table 1**).

**Table 1:** Antimicrobial susceptibilities and molecular typing of *Neisseria gonorrhoeae* isolates included in this study.

	Isolate ID							
	M009	M043	M107	M110	M111	M128	WHO F	WHO Y
Year of isolation	2018	2013	2015	2015	2015	2016	1991	2010
Source	Blood	Urethra	Urethra	Urethra	Urethra	Urethra	–	Urethra
Sequence type (NG-MAST)	4630	1407	338	338	1407	1407	3303	1407
**Minimum inhibitory concentration (mg/L)**								
Before induction of resistance to cefixime								
Azithromycin	0.125	0.25	0.125	0.125	0.25	0.25	0.125	1
Cefixime	0.016	0.125	0.016	0.016	0.125	0.125	0.004	2
Ceftriaxone	0.004	0.06	0.008	0.008	0.06	0.03	0.001	1
Ciprofloxacin	0.004	16	4	4	16	16	0.004	>32
Penicillin	0.5	2	8	8	4	2	0.03	1
Tetracycline	16	4	0.5	0.25	2	2	0.25	4
After induction of resistance to cefixime								
Azithromycin	0.06	0.25	0.25	0.125	0.125	0.25	0.125	0.06
Cefixime	0.5	1	2	2	8	16	0.5	>32
Ceftriaxone	0.06	0.5	0.25	0.25	4	4	0.06	32
Ciprofloxacin	0.004	16	4	4	16	32	0.004	32
Penicillin	0.5	4	8	0.5	8	8	0.06	8
Tetracycline	16	2	0.5	0.5	2	2	0.25	2

Breakpoints for cefixime: BrCAST, resistant > 0.125 mg/L; CLSI, resistant > 0.25 mg/L.

*N. gonorrhoeae* isolates were identified using a Vitek2 Compact instrument (BioMérieux S.A., Marcy l’Etoile, France). All isolates were stored at −80°C in casein-peptone soymeal-peptone broth (BD, Difco™, USA) containing 20% glycerol until further analysis.

### *In vitro* selection of resistance to cefixime

Gonococcal isolates were recovered from cryopreservation on chocolate agar plates (Laborclin, Parana, Brazil) and subcultured on the same medium. To induce resistance to cefixime, we subcultured a single colony on GC agar (Difco™, New Jersey, USA) supplemented with 1% Vitox (Oxoid™, Hampshire, UK) containing different concentrations of cefixime, starting from 0.002 mg/L. The colonies were subcultured on two GC plates: one containing the same concentration of cefixime and another with a higher concentration (1.5- to 2-fold each test). When the isolate grew on the higher-concentration plate, it was challenged to an even higher concentration of cefixime. All plates were incubated at 35 ± 1°C in 5% CO_2_ for 18 to 24 h. *In vitro* induction was considered complete when all isolates achieved stable growth at a cefixime concentration above the resistance breakpoint, according to guidelines of the Brazilian Committee on Antimicrobial Susceptibility Testing (BrCAST) (>0.125 mg/L, http://brcast.org.br/, accessed in March 2021). After resistance induction, all isolates were confirmed as *N. gonorrhoeae* using a Vitek2 Compact instrument and stored at −80°C in casein-peptone soymeal-peptone broth containing 20% glycerol.

### Antimicrobial susceptibility testing and molecular typing

Antimicrobial susceptibility testing was performed for six antimicrobial agents (**Table 1**) by agar dilution, as recommended by the Clinical and Laboratory Standards Institute. MICs were interpreted according to CLSI (CLSI, 2019) and BrCAST (available in http://brcast.org.br/) standards. *N. gonorrhoeae* WHO F, G, K, L, and M were used as controls (Unemo et al., 2016).

*N. gonorrhoeae* multi-antigen sequence typing (NG-MAST) was performed by PCR and sequencing of two *N. gonorrhoeae* genes (*porB* and *tbpB*), following the protocol developed by Martin et al. (2004). STs were identified by comparison with the NG-MAST database (http://www.ng-mast.net, accessed in 2018) (Golfetto, 2018).

### Genome sequencing and bioinformatics analysis

All isolates were sequenced, except WHO F and WHO Y before resistance induction. Genomic DNA was extracted using a PureLink™ Genomic DNA Mini Kit (Thermo Fisher Scientific Inc., Waltham, Massachusetts, USA) and quantified using the Qubit dsDNA BR Assay Kit (Thermo Fisher Scientific Inc). After quantification, DNA was diluted to 0.5 ng/µl. Illumina sequencing libraries were generated using an Illumina Nextera XT DNA Library Kit (Illumina Inc., San Diego, CA, USA). Whole-genome sequencing of paired-end libraries (PE, 2 × 250 bp) was performed using the Illumina MiSeq platform (Illumina Inc.).

Raw read quality was checked using FastQC version 0.11.8 (available in https://www.bioinformatics.babraham.ac.uk/projects/fastqc/), and quality-based trimming and filtering were performed using Trimmomatic version 0.39 (Bolger et al., 2014). Only paired-end reads from clinical isolates before cefixime resistance induction were assembled using SPAdes version 3.13.1 (Bankevich et al., 2012). Scaffold sequences were annotated using Prokka version 1.14.0 (Seemann, 2014). For reference strains, the genomes available at NCBI were used (WHO F, GenBank accession no. LT591897.1 and WHO Y, accession no. LT592161.1). *In silico* sequence typing was performed using MLST version 2.0 (https://cge.cbs.dtu.dk/services/MLST, accessed in January 2021).

Chromosomal and plasmid antibiotic resistance genes from assembled draft genomes of clinical isolates before resistance induction were predicted using the Comprehensive Antimicrobial Resistance Database (CARD) version 3.1.1 (https://card.mcmaster.ca/, accessed in January 2021) (McArthur et al., 2013). Mutations in antibiotic resistance-related genes were curated using Artemis version 18.0.0 (Carver et al., 2005).

For identification of cefixime resistance-induced mutations in the genome of gonococcal isolates, a single-nucleotide polymorphism (SNP)-calling pipeline was constructed by mapping reads from genomes sequenced after resistance induction onto the assembled genomes. This procedure was performed using BWA version 0.7.17-r1188 (Li and Durbin, 2009). SAMTools version 1.7 (Li et al., 2009) was used for manipulation of alignments. SNP calling was performed using BCFtools version 1.7 (Li, 2011), with a minimum Phred quality score of 23. The vcfutils.pl script was used to filter variants with at least 20-fold coverage depth. SNPs, nonsynonymous mutations, frameshift mutations caused by indels, and nonsense mutations resulting in premature stop codons in hypothetical proteins and coding sequences were annotated using SnpEff version 4 (Cingolani et al., 2012). A heatmap of mutations in known coding sequences in *N. gonorrhoeae* isolates after selection for resistance to cefixime (**Figure 2**) was constructed using R software version 4.0.2, and hierarchical clustering was applied using the default Ward2 algorithm (Murtagh and Legendre, 2014).

### Ethics approval

The Institutional Ethics Review Board of the University Hospital of the Federal University of Santa Catarina (HU/UFSC) approved this study under reference number CAAE 13727619.4.0000.0121.

### Nucleotide sequence accession numbers

Raw data and genome assemblies of all gonococcal isolates were deposited in the NCBI database under accession numbers SAMN26195797 to SAMN26195810.

## Results

### Molecular characterization of *N. gonorrhoeae* isolates

Draft genome sequences of isolates before selection for resistance to cefixime were used for genotypic characterization and identification of possible AMR determinants. Relevant genetic traits for antimicrobial resistance in *N. gonorrhoeae* are described in **Table 2**.

**Table 2:** Genetic traits of relevance for antimicrobial resistance in *Neisseria gonorrhoeae* isolates included in this study.

		M009	M043	M107	M110	M111	M128	WHO F	WHO Y
**Typing**	MLST	8161	1901	1588	1588	1901	1901	10934	1901
	NG-MAST	4630	1407	338	338	1407	1407	3303	1407
**Trait**	*penA* type	14.001	34.001	19.001	19.001	34.001	34.001	15.001	42.001
	PBP2 345insD	Yes	–	Yes	Yes	–	–	–	–
	*mtrR* promoter delA	–	Yes	–	–	Yes	Yes	–	Yes
	PBP1 L421P	Yes	Yes	Yes	Yes	Yes	Yes	–	Yes
	*rpsJ* codon V57M	Yes	Yes	Yes	Yes	Yes	Yes	–	Yes
	*gyrA* codon S91F	–	Yes	Yes	Yes	Yes	Yes	–	Yes
	*gyrA* codon D95G	–	Yes	–	–	Yes	Yes	–	Yes
	*parC* codon S87R	–	Yes	Yes	Yes	Yes	Yes	–	Yes
	*porB1b* codon G120K	NA	Yes	NA	NA	Yes	Yes	NA	Yes
	*porB1b* codon A121N	NA	Yes	NA	NA	Yes	Yes	NA	–
	*porB1b* codon A121D	NA	–	NA	NA	–	–	NA	Yes
	*bla*_TEM_ allele	–	–	TEM-1	TEM-1	–	–	–	–
	β-lactamase plasmid type	–	–	African	African	–	–	–	–
	*tet*(M) plasmid type	Dutch	–	–	–	–	–	–	–

NA, not applicable because these strains express PorB1a.

All isolates included in this study were found susceptible to the antimicrobials currently in use for the treatment of gonococcal infection in Brazil (ceftriaxone plus azithromycin) (Brasil, 2020). Of all isolates, M009, recovered from a blood sample, was the most susceptible to antimicrobials. This isolate showed mutations in PBP1 and PBP2, which are related to an increase in the MIC of penicillin. Furthermore, M009 was the only isolate to carry a TetM plasmid, which confers a high level of resistance to tetracycline.

Urethral discharge samples (M043, M107, M110, M111, and M128) showed similar resistance determinants according to ST profile. Samples belonging to ST1407/ST1901 (M043, M111, and M128) had a mosaic *penA*-34.001. The *penA* mosaic allele, generated by DNA transformation followed by recombination with commensal *Neisseria* spp., is associated with reduced susceptibility/resistance to ESCs, having a greater impact on the MIC of cefixime than that of ceftriaxone. The MIC of cefixime against these isolates (0.125 mg/L) revealed borderline resistance, according to BrCAST criteria (resistance = MIC > 0.125 mg/L); the MIC of ceftriaxone ranged from 0.03 to 0.06 mg/L. In addition to alterations in the primary ESC resistance mechanism, ST1407 isolates also exhibited alterations in PorB1b porin and MtrCDE efflux pump, influencing the MIC of ESCs; such alterations promote decreased influx and increased efflux of ESCs, respectively.

The MIC of ESCs for samples belonging to ST338/ST1588 (M107 and M110) was lower than that for ST1407. M107 and M110 harbored the *penA*-19.001 non-mosaic allele, and no alteration was found in the major determinants of ESC resistance. Of the six antimicrobials analyzed, M107 and M110 were only resistant to ciprofloxacin and penicillin. This pattern was associated with mutations in GyrA, ParC, and PBP1 as well as with the presence of β-lactamase-producing plasmid carrying *bla*_TEM-1_, which confers high resistance to penicillin but has no impact on the MIC of ESCs.

Isolates belonging to ST1407 and ST338 were resistant to ciprofloxacin because they contained mutations in GyrA and ParC. The 2-fold variation in MIC between STs may be explained by the extra GyrA mutation (D95G) observed in ST1407.

### Determination of MIC after resistance induction

**Figure 1:**
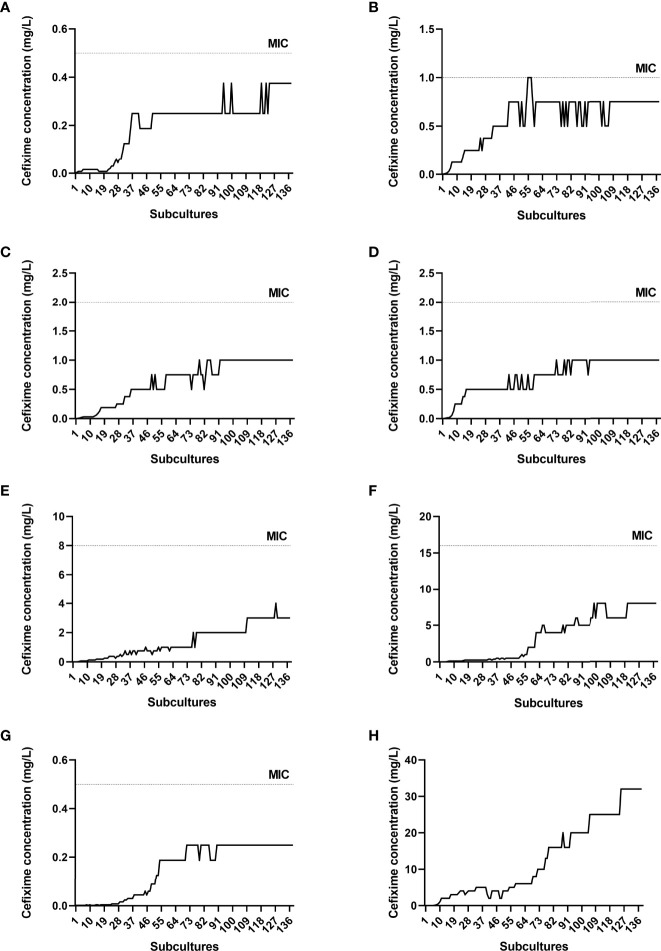
Progress curves during resistance evolution to cefixime. Dotted lines indicate minimum inhibitory concentration (MIC) values after induction. **(A)** M009, **(B)** M043, **(C)** M107, **(D)** M110, **(E)** M111, **(F)** M128, **(G)** WHO F, **(H)** WHO Y. The MIC for WHO Y is not shown (>32 mg/L).

*In vitro* resistance induction of *N. gonorrhoeae* isolates to cefixime was completed after 138 subcultures. **Figure 1** shows the progress curves during resistance evolution of each isolate. It can be noted that some isolates took longer to grow (M128 and WHO F), whereas others adapted more quickly to cefixime.

There was an increase in cefixime MIC, such that all susceptible isolates became resistant according to BrCAST (resistant = MIC > 0.125 mg/L) and CLSI (resistant = MIC > 0.25 mg/L) criteria (**Table 1**). The MIC for M043 showed a minor variation, with an 8-fold increase compared with the initial MIC, and that for M128 increased 128-fold. The MIC against the unique gonococcus isolate WHO Y, which was initially resistant to cefixime, was greater than 32 mg/L after induction.

The MICs of cefixime, ceftriaxone, and penicillin differed after resistance induction, whereas those of azithromycin, ciprofloxacin, and tetracycline remained stable. We observed a reduction in the MIC of penicillin against M009 and M110; thus, these isolates, which were initially resistant, became sensitive to the antimicrobial. M110, which had a plasmid carrying *bla*_TEM-1_, lost the plasmid at the end of subculturing, resulting in a penicillin MIC of 0.5 mg/L.

### Mapping of mutations and gene function in cefixime-resistant isolates postselection

Subculturing of isolates with cefixime resulted in mutations in genes related or not to ESC resistance. SNPs in the main resistance determinant (*penA*) were observed in five isolates: WHO F and M009, which do not have the *penA* mosaic form, and M043, M111, and M128, which belong to ST1407. M111 acquired one (A488V) and M128 acquired four (A318V, R356H, N506H, and G546A) novel mutations in *penA*. These isolates exhibited the highest cefixime MIC. **Table 3** shows the changes in amino acids of isolates as compared with the reference M32091 (GenBank M32091.1).

**Table 3:** Amino acid sequence alignment of penicillin-binding protein 2 of *Neisseria gonorrhoeae* isolates before and after selection for resistance to cefixime.

			Amino Acid Position According to the Reference Strain M32091																																																																																							
			1		1		1		1		2		2		2		2		2		2		2		2		2		2		2		2		2		2		2		2		2		2		2		2		2		2		3		3		3		3		3		3		3		3		3		3		3		3		3		3		3		3				3	3
			0		6		7		9		0		0		0		0		0		1		1		2		2		3		3		3		4		5		5		7		7		8		8		8		9		9		1		1		1		1		2		2		2		2		3		3		3		3		4		4		4		4				5	5
			1		0		3		6		1		2		3		4		7		3		4		7		8		0		3		5		9		6		9		1		9		1		5		8		1		3		1		2		6		8		3		6		8		9		0		1		2		5		1		2		3		5				4	6
M32091		Reference strain	D		V		N		L		Y		G		E		D		E		R		Q		K		A		Q		K		I		E		E		Q		R		A		T		D		R		R		D		A		I		V		A		A		T		L		N		E		R		L		Q		P		S		P		R		–		S	R
WHO F		Unemo et al. (2016)	.		.		.		.		.		.		.		.		.		.		.		.		.		.		.		.		.		.		.		.		.		.		.		.		.		.		.		.		.		.		.		.		.		.		.		.		.		.		.		.		.		.		–		.	.
WHO F*		This study	.		.		.		.		.		.		.		.		.		.		.		.		.		.		.		.		.		.		.		.		.		.		.		.		.		.		.		.		.		.		.		.		.		.		.		.		.		.		.		.		.		.		–		.	.
WHO Y		Unemo et al. (2011)	E		A		S		.		H		A		G		E		.		.		E		.		.		.		.		.		.		.		.		.		V		.		E		K		Q		.		.		M		T		.		S		V		A		T		D		T		F		L		S		A		T		Q		–		T	.
WHO Y*		This study	E		A		S		.		H		A		G		E		.		.		E		.		.		.		.		.		.		.		.		.		V		.		E		K		Q		.		.		M		T		.		S		V		A		T		D		T		F		L		S		A		T		Q		–		T	.
M009		This study	.		.		.		.		.		.		.		.		.		.		.		.		.		.		.		.		.		.		.		.		.		.		.		.		.		.		.		.		.		.		.		.		.		.		.		.		.		.		.		.		.		.		D		.	.
M009*		This study	.		.		.		.		.		.		.		.		.		.		.		.		.		.		.		.		.		.		.		.		.		.		.		.		.		.		V		.		.		.		.		.		.		.		.		.		.		.		.		.		.		.		D		.	.
M043		This study	E		A		S		.		H		A		G		E		.		.		E		.		.		.		.		.		.		.		.		.		V		.		E		K		Q		.		.		M		T		.		S		V		A		T		D		T		F		L		S		A		T		Q		–		T	.
M043*		This study	E		A		S		.		H		A		G		E		.		.		E		.		.		.		.		.		.		.		.		.		V		.		E		K		Q		.		.		M		T		.		S		V		A		T		D		T		F		L		S		A		T		Q		–		T	.
M107		This study	.		.		.		.		.		.		.		.		.		.		.		.		.		.		.		.		.		.		.		.		.		.		.		.		.		.		.		.		.		.		.		.		.		.		.		.		.		.		.		.		.		.		D		.	.
M107*		This study	.		.		.		.		.		.		.		.		.		.		.		.		.		.		.		.		.		.		.		.		.		.		.		.		.		.		.		.		.		.		.		.		.		.		.		.		.		.		.		.		.		.		D		.	.
M110		This study	.		.		.		.		.		.		.		.		.		.		.		.		.		.		.		.		.		.		.		.		.		.		.		.		.		.		.		.		.		.		.		.		.		.		.		.		.		.		.		.		.		.		D		.	.
M110*		This study	.		.		.		.		.		.		.		.		.		.		.		.		.		.		.		.		.		.		.		.		.		.		.		.		.		.		.		.		.		.		.		.		.		.		.		.		.		.		.		.		.		.		D		.	.
M111		This study	E		A		S		.		H		A		G		E		.		.		E		.		.		.		.		.		.		.		.		.		V		.		E		K		Q		.		.		M		T		.		S		V		A		T		D		T		F		L		S		A		T		Q		–		T	.
M111*		This study	E		A		S		.		H		A		G		E		.		.		E		.		.		.		.		.		.		.		.		.		V		.		E		K		Q		.		.		M		T		.		S		V		A		T		D		T		F		L		S		A		T		Q		–		T	.
M128		This study	E		A		S		.		H		A		G		E		.		.		E		.		.		.		.		.		.		.		.		.		V		.		E		K		Q		.		.		M		T		.		S		V		A		T		D		T		F		L		S		A		T		Q		–		T	.
M128*		This study	E		A		S		.		H		A		G		E		.		.		E		.		.		.		.		.		.		.		.		.		V		.		E		K		Q		.		V		M		T		V		S		V		A		T		D		T		F		L		S		A		T		Q		–		T	H

				3		3		3		3		3		3		4		4		4		4		4		4		4		4		4		4		4		4		4		4		4		4		4		4		4		4		5		5		5		5		5		5		5		5		5		5		5		5		5		5		5		5				5
				7		7		7		7		8		8		0		0		0		0		1		1		3		4		4		5		6		6		6		6		6		7		8		8		8		8		0		0		0		1		1		1		3		4		4		4		4		5		5		5		5		6				7
				3		5		6		7		5		8		0		3		6		9		1		2		7		3		7		7		1		2		4		8		9		2		0		3		5		8		1		4		6		0		2		6		4		1		5		6		9		1		2		5		6		6				4
M32091	Reference strain			R		G		A		E		E		I		T		L		N		R		R		P		A		V		L		Q		I		F		E		R		E		N		P		T		T		A		A		F		N		A		N		A		T		H		G		G		A		P		P		K		I		I		–		A
WHO F	Unemo et al. (2016)			.		.		.		.		.		.		.		.		.		.		.		.		.		.		.		.		.		.		.		.		.		.		.		.		.		.		.		.		.		.		.		.		.		N		.		.		.		.		.		.		.		.		–		.
WHO F*	This study			.		.		.		.		.		.		.		.		.		.		.		.		.		.		.		.		.		.		.		.		.		.		.		.		.		.		.		.		.		.		.		.		.		N		.		.		.		S		.		.		.		.		–		.
WHO Y	Unemo et al. (2011)			M		T		P		K		D		V		.		.		S		.		Q		K		V		E		V		K		V		I		A		K		K		E		A		.		.		.		P		L		.		V		Y		.		.		N		S		.		.		.		.		.		.		.		–		.
WHO Y*	This study			M		T		P		K		D		V		.		.		S		.		Q		K		V		E		V		K		V		I		A		K		K		E		A		.		.		.		P		L		.		V		Y		.		.		N		S		.		.		.		.		.		.		.		–		.
M009	This study			.		.		.		.		.		.		.		.		.		.		.		.		.		.		.		.		.		.		.		.		.		.		.		.		.		.		.		L		.		V		.		G		.		N		.		.		.		.		.		.		.		.		–		.
M009*	This study			.		.		.		.		.		.		.		.		.		.		.		.		.		.		.		.		.		.		.		.		.		.		.		.		.		.		.		L		.		V		.		G		.		N		.		.		.		.		.		.		.		.		–		.
M043	This study			M		T		P		K		D		V		.		.		S		.		Q		K		V		E		V		K		V		I		A		K		K		E		A		.		.		.		.		L		.		V		Y		.		.		N		S		.		.		.		.		.		.		.		–		.
M043*	This study			M		T		P		K		D		V		.		.		S		.		Q		K		V		E		V		K		V		I		A		K		K		E		A		.		.		.		.		L		.		V		Y		.		.		N		S		.		.		S		.		.		.		.		–		.
M107	This study			.		.		.		.		.		.		.		.		.		.		.		.		.		.		.		.		.		.		.		.		.		.		.		.		.		.		.		L		.		V		.		G		.		N		.		.		.		.		.		.		.		V		N		V
M107*	This study			.		.		.		.		.		.		.		.		.		.		.		.		.		.		.		.		.		.		.		.		.		.		.		.		.		.		.		L		.		V		.		G		.		N		.		.		.		.		.		.		.		V		N		V
M110	This study			.		.		.		.		.		.		.		.		.		.		.		.		.		.		.		.		.		.		.		.		.		.		.		.		.		.		.		L		.		V		.		G		.		N		.		.		.		.		.		.		.		V		N		V
M110*	This study			.		.		.		.		.		.		.		.		.		.		.		.		.		.		.		.		.		.		.		.		.		.		.		.		.		.		.		L		.		V		.		G		.		N		.		.		.		.		.		.		.		V		N		V
M111	This study			M		T		P		K		D		V		.		.		S		.		Q		K		V		E		V		K		V		I		A		K		K		E		A		.		.		.		.		L		.		V		Y		.		.		N		S		.		.		.		.		.		.		.		–		.
M111*	This study			M		T		P		K		D		V		.		.		S		.		Q		K		V		E		V		K		V		I		A		K		K		E		A		.		.		V		.		L		.		V		Y		.		A		N		S		.		.		S		.		.		.		.		–		.
M128	This study			M		T		P		K		D		V		.		.		S		.		Q		K		V		E		V		K		V		I		A		K		K		E		A		.		.		.		.		L		.		V		Y		.		.		N		S		.		.		.		.		.		.		.		–		.
M128*	This study			M		T		P		K		D		V		.		.		S		.		Q		K		V		E		V		K		V		I		A		K		K		E		A		.		.		.		.		L		H		V		Y		.		.		N		S		A		.		S		.		.		.		.		–		.

Amino acid changes are represented by a single capital letter. An asterisk indicates isolates after selection. Amino acid modifications after cefixime induction are highlighted in green. Amino acid modifications not reported in the literature are highlighted in blue.

**Figure 2:**
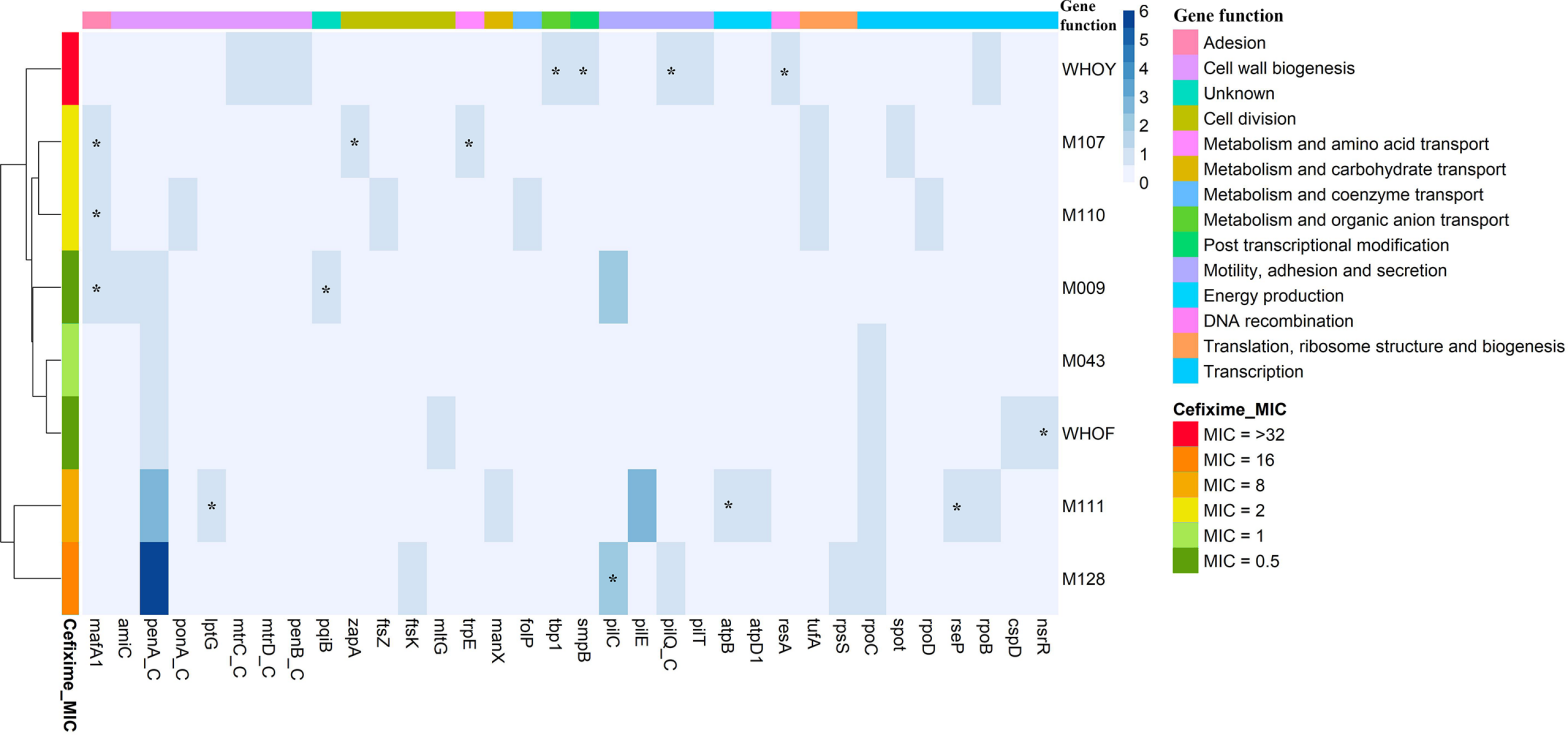
Heatmap of mutations in known coding sequences in *Neisseria gonorrhoeae* isolates after selection for resistance to cefixime and description of affected gene functions. Isolates and genes are presented in rows and columns, respectively. The blue color scale represents the amount of acquired mutations. The left sidebar represents the minimum inhibitory concentration (MIC) of cefixime (mg/L). The top bar indicates the function of each gene. _C indicates determinants related to extended-spectrum cephalosporin resistance; asterisk indicates genes that showed a frameshift.

In addition to mutations in *penA*, we observed nonsynonymous, frameshift, and stop codon mutations in genes contributing to ESC resistance and in other genes. **Table S1** shows all mutations in coding genes that emerged in the isolates of this study. **Figure 2** shows a heatmap of all mutations in known coding sequences. Several metabolic pathways were affected by cefixime induction. Two of the most affected pathways were cell wall biogenesis and cell division. Pilus formation genes (*pil* genes), associated with motility, adhesion, and secretion, were also affected at the DNA level. The other mutated genes were related to DNA recombination; energy production; metabolism/transport of amino acids, organic ions, carbohydrates, and coenzymes; post-transcriptional modifications; and translation. One gene was associated with an unknown function.

A significant variation in mutation patterns was identified after induction with cefixime. No gene was altered in all isolates. M111 and M128 were grouped for having the highest number of mutations in *penA*. M107 and M110, which belong to ST338, formed a group. The other group was formed by WHO F isolates, M009 and M043, which exhibited the lowest cefixime MIC values.

WHO Y, which did not show any mutation in *penA* but exhibited an increase in the MIC of ESCs, suffered mutations in some resistance genes, including porin PorB1b (A26V), MtrC (S23P), and MtrD (M570K). Mutations in PilQ were observed in M128 (G590D) and WHO Y (guanine insertion between nucleotides 1566 and 1567), resulting in a frameshift. Interestingly, M107 and M110 did not acquire mutations in the main determinants of resistance to ESCs. Only M110 acquired an SNP in *ponA* (PBP1), which resulted in an A615V amino acid change in this protein. Nevertheless, we observed alterations in genes involved in cellular division. M107 acquired a guanine insertion between nucleotides 299 and 300, resulting in a frameshift in *zapA*, and M110 acquired the A318V mutation in FtsZ.

## Discussion

In this study, we assessed DNA alterations in *N. gonorrhoeae* isolates after cefixime exposure. WHO F, WHO Y, one *N. gonorrhoeae* isolate from blood culture, and the two most prevalent STs in Brazil were used (Golparian et al., 2020).

The MIC of cefixime increased above the breakpoint (as determined by CLSI and BrCAST criteria) for all isolates postselection, indicating that isolates acquired resistance to this antimicrobial. A study conducted by Gong et al. (2016) subjected five WHO reference strains of *N. gonorrhoeae* and one clinical isolate to resistance induction using ceftriaxone. Only the reference strain WHO D acquired resistance after 136 subcultures, increasing the MIC to 1.0 mg/L (initial MIC = 0.016 mg/L). WHO D possessed non-mosaic *penA*, adenine deletion in the *mtrR* promoter region, and L421P amino acid change in PBP1. Furthermore, Gong et al. (2016) found that, of all frequently reported genes associated with ceftriaxone resistance in *N. gonorrhoeae*, only *penA* was mutated in WHO D (A501V). Although Gong et al. (2016) used liquid cultures to induce resistance, this finding may demonstrate a low ability of ceftriaxone to induce resistance *in vitro* in *N. gonorrhoeae* compared with cefixime.

The PBP2 protein is part of class B penicillin-binding proteins, which catalyze the last step of peptidoglycan formation by crosslinking peptides (Ghuysen, 1991). The active site of PBP2, to which β-lactam antimicrobials bind *via* an acylation reaction, contains three conserved motifs: SxxK, which comprises the amino acids Ser-310 and Lys-313 (α2 helix); SxN, which contains Ser-362 and Asn-364 (between helices α4 and α5); and KTG, comprising amino acids Lys-497, Tre-498, and Gly-499 (β3 sheet) (Powell et al., 2009). The presence of mosaic *penA* is associated with the emergence of mutations close to these motifs. Such mutations alter protein conformation, impairing the binding of antimicrobials or preventing conformational changes that are necessary for acylation by β-lactams (Osaka et al., 2008; Powell et al., 2009). The first isolate showing a high level of ESC resistance was reported in Japan (H041) (Ohnishi et al., 2011; Unemo and Shafer, 2014). Ceftriaxone and cefixime had MIC values of 2.0 and 4.0 mg/L, respectively, against the isolate. Later studies demonstrated that A311V (observed here in M009 and M128), V316P, and T483S mutations resulted in a significant increase in the MIC values of cefixime and ceftriaxone (Tomberg et al., 2013; Unemo and Shafer, 2014). The A311V alteration is present in most isolates reported with ESC resistance and was found to emerge in M009 and M128 after resistance induction, which probably contributed to the increase in MIC values for these isolates.

Interestingly, the isolates for which MIC values were highest showed amino acid changes not previously described. M128 showed four amino acid changes (A318V, R356H, N506H, and G546A) and M111 exhibited the A488V mutation, also not previously described. These mutations appear close to conserved motifs that are part of the PBP2 active site. Furthermore, the presence of T534A mutations in M111 and A311V mutation in M128, associated with the P551S mutation, whose simultaneous occurrence in isolates with ESC resistance is not common, may be responsible for the high MIC values. It is noteworthy that, in addition to the acquired mutations in *penA*, these isolates previously exhibited mutations in PorB1b and the *mtrR* promoter region, which probably helped to increase the MIC. The contribution of PBP2 mutations in M111 and M128 after induction, as well as the ability of the microorganism to face these changes, still needs to be investigated.

The amino acid change T534A, found in this study only in M111, was described in Austria in an isolate belonging to ST1407 with mosaic *penA*-34.001. The isolate led to treatment failure with the use of cefixime. Transformation experiments confirmed that only this new mutation was responsible for ESC resistance (Unemo et al., 2011). The Austrian isolate also had a deletion in the *mtrR* promoter region, G120K and A121N mutations in PorB, and L421P mutation in PBP1, as did M111 in the current study. However, the Austrian isolate had lower MICs than M111 (1.0 mg/L for cefixime and 0.5 mg/L for ceftriaxone), suggesting that other mutations might have contributed to the increase in MIC in M111.

P551S was the most frequent PBP2 mutation in isolates postselection. This mutation was observed in WHO F and isolates belonging to ST1407. The P551S mutation is more frequent in isolates lacking the mosaic *penA*. Kinetic analysis of β-lactam acylation at the active site of PBP2 indicated that two mutations (P551S and F504L) are responsible for reducing the acylation index, which minimizes the affinity for PBP2–antimicrobial complex formation (Powell et al., 2009). These mutations are usually found in non-mosaic type PBP2; however, they emerged in *penA*-34.001 isolates after antimicrobial selection in the current study.

In addition to the occurrence of *penA* mutations in most of the isolates postselection, we observed mutations in genes associated or not with ESC resistance. Various metabolic pathways were affected by selection with cefixime. Given that cefixime acts by inhibiting bacterial cell wall formation, most of the mutated genes are involved in cell wall biogenesis and cell division. Thirteen genes exhibited frameshift mutations, which may lead to the formation of defective proteins. Because we did not analyze gene expression, it was not possible to affirm whether these pathways were over- or underexpressed. In addition to a possible association with ESC resistance, the variety of genes found with mutations in this study may represent the acquisition of compensatory mutations. Vincent et al. (2018) demonstrated the acquisition of this type of mutation in *acnB* gene in an *N. gonorrhoeae* isolate containing mosaic *penA*. In fact, compensatory mutations may emerge in *N. gonorrhoeae* ESC-resistant to improve their fitness, thus being able to maintain and disseminate the lineage.

The mutations associated with decreased permeability of antimicrobials in *N. gonorrhoeae* are amino acid substitutions at positions 120 and 121 of PorB1b (Olesky et al., 2002; Unemo and Shafer, 2014). The impact of these mutations on cephalosporin resistance in clinical isolates only became apparent with overexpression of the MtrCDE efflux pump (Olesky et al., 2006; Shafer and Folster, 2006; Unemo and Shafer, 2014). Such an effect causes a decrease in antimicrobial input and an increase in output. The PorB1b mutation observed in the current study in WHO Y had not yet been described or related to an increase in the MIC of ESCs. Therefore, complementary experiments are needed to prove whether the mutation influences resistance to ESCs.

WHO Y also exhibited mutations in MtrC and MtrD. MtrD, together with membrane fusion (MtrC) and outer membrane channel (MtrE) proteins, exports antimicrobials out of the bacterial cell (Hagman et al., 1995; Delahay et al., 1997). Mutations in clinical isolates that cause resistance to various antimicrobials through this efflux pump are associated with changes in its repressor (*mtrR*) (Hagman et al., 1995; Zarantonelli et al., 1999; Unemo and Shafer, 2014). Such changes generally comprise adenine deletion in the *mtrR* promoter region (found here in ST1407 isolates) and the G45D amino acid change in MtrR (Hagman et al., 1995; Zarantonelli et al., 1999; Unemo and Shafer, 2014). In 2018, it was experimentally found that the presence of a series of mutations in the Mtr efflux pump was associated with azithromycin resistance (Wadsworth et al., 2018). Thus, similar to what was observed for *penA*, this mosaic-like structure was acquired from other *Neisseria* spp. (Johnson et al., 2003; McLean et al., 2004). The protein MtrD contains an antimicrobial binding site; in a recent study, it was shown that the amino acid M570 occurs at this site but is conserved in the *Escherichia coli* efflux system (Lyu et al., 2020). The mutations S23P (MtrC) and M570K (MtrD) founded in WHO Y are not seen in isolates with *mtr* mosaic (Rouquette-Loughlin et al., 2018; Wadsworth et al., 2018). Point mutations in PorB and MtrC/D, associated with mutations in WHO Y before induction, might have contributed to the increase in MIC.

WHO Y and M128 showed mutations in PilQ. M128 exhibited the G590D mutation, whereas WHO Y exhibited an insertion of guanine between nucleotides 1566 and 1567, which resulted in a frameshift. PilQ belongs to a family of outer membrane proteins known as secretins. PilQ is the only secretin in *N. gonorrhoeae*; it plays a role in the formation of channels or pores through which the pilus is externalized (Drake and Koomey, 1995; Tønjum et al., 1998). Specific mutations in PilQ (e.g., E666K mutation) can lead to altered permeability of antimicrobials such as penicillin and tetracycline but do not seem to affect the MIC of ESCs (Ropp et al., 2002; Zhao et al., 2005; Zhao et al., 2009; Unemo and Shafer, 2014). A frameshift mutation in *pilQ* was observed *in vitro* by Johnson et al. (2014) and found to result in a 10-fold increase in ceftriaxone MIC. Thus, the mutations in PilQ in WHO Y might have contributed to the increase in cefixime and ceftriaxone MICs. Given that the adhesion of gonococcus to epithelial cells is mediated by type IV pili, the expression of this protein is essential for *N. gonorrhoeae* pathogenesis. Thus, the frameshift in WHO Y might have transformed the isolate into non-competent for infection.

Interestingly, the MIC of cefixime for M107 and M110 increased 125-fold after induction, whereas that of ceftriaxone increased 31.25-fold. Although this study did not focus on mutations in intergenic regions, a search for changes in the *mtrR* promoter region was performed, and no mutations were found in M107 or M110. These isolates did not have mutations in the main ESC resistance determinants before induction. After induction, M110 acquired a mutation in *ponA*, which resulted in an A615V amino acid change in PBP1. In clinical isolates, the contribution of PBP1 to higher ESC MIC values was only observed when associated with other determinants, *mtrR* and *penA*, given that ESCs have a higher affinity for PBP2 (Zhao et al., 2009). Therefore, this change alone could not explain the increase in the MIC for M110. Gong et al. (2016) found that a mutation in the subunit of a cell division-related ABC transporter (*ftsX*) was responsible for an increase in ceftriaxone MIC. In M107 and M110, mutations were observed in *zapA* and *ftsZ*, respectively, two genes involved in cell division. *ftsZ* is part of a group of genes involved in both cell division and synthesis of peptidoglycan, which initiates cell division by forming a polymer called the Z ring. This structure gives rise to new bacterial cells (Ayala et al., 1994). ZapA is a regulator of *ftsZ*, which helps in the initial formation and posterior stabilization of the polymer (Low et al., 2004). Deletion of *zapA* in *E. coli* did not result in a phenotypic change but was shown to be lethal when FtsZ levels were reduced (Johnson et al., 2004). Thus, the frameshift observed in *zapA* in the M107 isolate, associated with selective environmental pressure of the antimicrobial, which inhibits cell wall formation, may favor bacterial division. As this mechanism has not been observed in clinical isolates circulating in the world, further studies are necessary to elucidate the mechanisms that caused resistance in these isolates. It is suggested that such mechanisms are more involved with resistance to cefixime than to ceftriaxone.

In 2020, a study identified different alleles of RNA polymerase associated with ceftriaxone resistance (Palace et al., 2020). They evidenced that clinical isolates of *N. gonorrhoeae* from different genetic backgrounds can acquire high-level resistance for ceftriaxone through RNA polymerase mutations. In this study, we observed that mutations in RNA polymerase (*rpoB*, *rpoC*, and *rpoD*) arose in six out of eight isolates postselecion, including isolates mosaic *penA*-34.001 and nonmosaic.

In the current study, we observed the ability of the antimicrobial cefixime to induce resistance in *N. gonorrhoeae* isolates *in vitro*. Although not currently used in Brazil, cefixime can serve as a marker of resistance, given that its MIC is generally higher than that of ceftriaxone. It was interesting to note that different STs acquired mutation resistance to ESCs after exposure to cefixime by different mechanisms. Isolates with *penA* mosaic were more likely to develop mutations in the *penA* gene itself. These findings reinforce the importance of the gonococcal antimicrobial resistance surveillance program in Brazil, given the changes in treatment recommendations in 2017, and the nationwide prevalence of STs that can acquire resistance to these antimicrobials.

## Data Availability Statement

The datasets presented in this study can be found in online repositories. The names of the repository/repositories and accession number(s) can be found below:

https://www.ncbi.nlm.nih.gov/, SAMN26195797,

https://www.ncbi.nlm.nih.gov/, SAMN26195798

https://www.ncbi.nlm.nih.gov/, SAMN26195799

https://www.ncbi.nlm.nih.gov/, SAMN26195800

https://www.ncbi.nlm.nih.gov/, SAMN26195801

https://www.ncbi.nlm.nih.gov/, SAMN26195802

https://www.ncbi.nlm.nih.gov/, SAMN26195803

https://www.ncbi.nlm.nih.gov/, SAMN26195804

https://www.ncbi.nlm.nih.gov/, SAMN26195805

https://www.ncbi.nlm.nih.gov/, SAMN26195806

https://www.ncbi.nlm.nih.gov/, SAMN26195807

https://www.ncbi.nlm.nih.gov/, SAMN26195808

https://www.ncbi.nlm.nih.gov/, SAMN26195809

https://www.ncbi.nlm.nih.gov/, SAMN26195810.

## Author Contributions

MAS and MB conceived and designed the study. MCS assisted with sample isolation and identification. MAS, FB, JM, HM, HG, and JP conducted lab experiments. MAS, DM, and JP carried out genome analysis. MRS assisted in bioinformatics analysis. MAS, JP, and MB interpreted the data and wrote the manuscript. MB supervised the study. All authors edited, commented, and approved the final version of the manuscript. All authors contributed to the article and approved the submitted version.

## Funding

The present study was supported by the Ministry of Health of Brazil, through its Secretariat for Health Surveillance/Department of Diseases of Chronic Condition and Sexually Transmitted Infections.

## Acknowledgments

We thank the Ministry of Health of Brazil and its Diseases of Chronic Condition and Sexually Transmitted Infections for offering support in *N. gonorrhoeae* isolate identification and MIC determination.

## Conflict of Interest

The authors declare that the research was conducted in the absence of any commercial or financial relationships that could be construed as a potential conflict of interest.

## Publisher’s Note

All claims expressed in this article are solely those of the authors and do not necessarily represent those of their affiliated organizations, or those of the publisher, the editors and the reviewers. Any product that may be evaluated in this article, or claim that may be made by its manufacturer, is not guaranteed or endorsed by the publisher.

## References

[B1] Allen V. G. Mitterni L. Seah C. Rebbapragada A. Martin I. E. Lee C.. (2013). Neisseria gonorrhoeae treatment failure and susceptibility to cefixime in Toronto, Canada. Am. Med. Assoc. 309, 163–170. doi: 10.1001/jama.2012.176575 23299608

[B2] Ameyama S. Onodera S. Takahata M. Minami S. Maki N. Endo K.. (2002). Mosaic-like structure of penicillin-binding protein 2 gene (penA) in clinical isolates of neisseria gonorrhoeae with reduced susceptibility to cefixime. Antimicrob Agents Chemother 46, 3744–3749. doi: 10.1128/AAC.46.12.3744-3749.2002 12435671 PMC132769

[B3] (2020) Brazilian Committee on antimicrobial susceptibility testing v. 8.0. Available at: http://brcast.org.br/.

[B4] Ayala J. A. Garrido T. de Pedro M. A. Vicente M. (1994). Molecular biology of bacterial septation. In Ghuysen J. M. Hakenbeck R. (ed.) New Comprehensive Biochemistry Elsevier 27, 73–101. doi: 10.1016/S0167-7306(08)60408-1

[B5] Bankevich A. Nurk S. Antipov D. Gurevich A. A. Dvorkin M. Kulikov A. S.. (2012). SPAdes: A new genome assembly algorithm and its applications to single-cell sequencing. J. Comput. Biol. 19, 455–477. doi: 10.1089/cmb.2012.0021 22506599 PMC3342519

[B6] Barbee L. A. Nayak S. U. Blumer J. L. O’Riordan M. A. Gray W. Zenilman J. M.. (2018). A phase 1 pharmacokinetic and safety study of extended-duration, high-dose cefixime for cephalosporin-resistant neisseria gonorrhoeae in the pharynx. Sexually Transmitted Dis. 45, 677–683. doi: 10.1097/OLQ.0000000000000844 29624558

[B7] Bazzo M. L. Golfetto L. Gaspar P. C. Pires A. F. Ramos M. C. Franchini M.. (2018). First nationwide antimicrobial susceptibility surveillance for neisseria gonorrhoeae in Brazil, 2015-16. J. Antimicrob Chemother 73, 1854–1861. doi: 10.1093/jac/dky090 29635367

[B8] Bolger A. M. Lohse M. Usadel B. (2014). Trimmomatic: A flexible trimmer for illumina sequence data. Bioinformatics 30, 2114–2120. doi: 10.1093/bioinformatics/btu170 24695404 PMC4103590

[B9] Brasil (2015). Protocolo clínico e diretrizes terapêuticas (Brasília: Brasil: Atenção integral às pessoas com Infecções Sexualmente Transmissíveis), 119p. Available at: https://bvsms.saude.gov.br/bvs/publicacoes/protocolo_clinico_diretrizes_terapeutica_atencao_integral_pessoas_infeccoes_sexualmente_transmissiveis.pdf.

[B10] Brasil (2020). Protocolo clínico e diretrizes terapêuticas para atenção integral às pessoas com infecções sexualmente transmissíveis (Brasília: Brasil), 250p. Available at: http://www.aids.gov.br/pt-br/pub/2015/protocolo-clinico-e-diretrizes-terapeuticas-para-atencao-integral-pessoas-com-infeccoes.

[B11] Cámara J. Serra J. Ayats J. Bastida T. Carnicer-Pont D. Andreu A.. (2012). Molecular characterization of two high-level ceftriaxone-resistant neisseria gonorrhoeae isolates detected in Catalonia, Spain. J. Antimicrob Chemother 67, 1858–1860. doi: 10.1093/jac/dks162 22566592

[B12] Carver T. J. Rutherford K. M. Berriman M. Rajandream M. A. Barrell B. G. Parkhill J. (2005). ACT: The Artemis comparison tool. Bioinformatics 21, 3422–3423. doi: 10.1093/bioinformatics/bti553 15976072

[B13] Chisholm S. A. Mouton J. W. Lewis D. A. Nichols T. Ison C. A. Livermore D. M. (2010). Cephalosporin MIC creep among gonococci: Time for a pharmacodynamic rethink? J. Antimicrob Chemother 65, 2141–2148. doi: 10.1093/jac/dkq289 20693173

[B14] Cingolani P. Platts A. Wang L. L. Coon M. Nguyen T. Wang L.. (2012). A program for annotating and predicting the effects of single nucleotide polymorphisms, SnpEff: SNPs in the genome of drosophila melanogaster strain w1118; iso-2; iso-3. Fly (Austin) 6, 80–92. doi: 10.4161/fly.19695 22728672 PMC3679285

[B15] CLSI (2019). M100 performance standards for antimicrobial susceptibility testing. J. Serv. Marketing 8, 1–320.

[B16] Delahay R. M. Robertson B. D. Balthazar J. T. Shafer W. M. Isonl C. A. (1997). Involvement of the gonococcal MtrE protein in the resistance of neisseria gonorrhoeae to toxic hydrophobic agents. Microbiology 143, 2127–2133. doi: 10.1099/00221287-143-7-2127 9245802

[B17] Drake S. L. Koomey M. (1995). The product of the pilQ gene is essential for the biogenesis of type IV pill in neisseria gonorrhoeae. Molecular Microbiol. 18, 975–986. doi: 10.1111/j.1365-2958.1995.18050975.x 8825101

[B18] Ghuysen J.-M. (1991). SERINE b-LACTAMASES AND PENICILLIN-BINDING PROTEINS. Annu. Rev. Microbiol. 45, 37–67. doi: 10.1146/annurev.mi.45.100191.000345 1741619

[B19] Gill M. J. Simjee S. Al-Hattawi K. Robertson B. D. Easmon C. S. F. Ison C. A. (1998). Gonococcal resistance to-lactams and tetracycline involves mutation in loop 3 of the porin encoded at the penB locus. Antimicrob. Agents Chemother. 42, 2799–2803. doi: 10.1128/AAC.42.11.2799 9797206 PMC105946

[B20] Golfetto L. (2018). Caracterização molecular e determinação do perfil de resistência de isolados clínicos de neisseria gonorrhoeae circulantes na grande florianópolis: série histórica 2008-2016 ([Florianópolis (SC)]: Universidade Federal de Santa Catarina).

[B21] Golparian D. Bazzo M. L. Golfetto L. Gaspar P. C. Schörner M. A. Benzaken A. S.. (2020). Genomic epidemiology of neisseria gonorrhoeae elucidating the gonococcal antimicrobial resistance and lineages/sublineages across Brazil 2015–16. J. Antimicrob Chemother 75, 3163–3172. doi: 10.1093/jac/dkaa318 32785692

[B22] Golparian D. Shafer W. M. Ohnishi M. Unemo M. (2014). Importance of multidrug efflux pumps in the antimicrobial resistance property of clinical multidrug-resistant isolates of neisseria gonorrhoeae. Antimicrob Agents Chemother 58, 3556–3559. doi: 10.1128/AAC.00038-14 24733458 PMC4068427

[B23] Gong Z. Lai W. Liu M. Hua Z. Sun Y. Xu Q.. (2016). Novel genes related to ceftriaxone resistance found among ceftriaxone-resistant neisseria gonorrhoeae strains selected *in vitro* . Antimicrob Agents Chemother 60, 2043–2051. doi: 10.1128/AAC.00149-15 26787702 PMC4808169

[B24] Hagman K. E. Pan W. Spratt B. G. Balthazar J. T. Judd R. C. Shafer W. M. (1995). Resistance of neisseria gonorrhoeae to antimicrobial hydrophobic agents is modulated by the mtrRCDE efflux system. Microbiology 141, 611–622. doi: 10.1099/13500872-141-3-611 7711899

[B25] Hagman K. E. Shafer W. M. (1995). Transcriptional control of the mtr efflux system of neisseria gonorrhoeae. J. Bacteriol. 177, 4162–4165. doi: 10.1128/jb.177.14.4162-4165.1995 7608095 PMC177154

[B26] Ito M. Deguchi T. Mizutani K. S. Yasuda M. Yokoi S. Ito S. I.. (2005). Emergence and spread of neisseria gonorrhoeae clinical isolates harboring mosaic-like structure of penicillin-binding protein 2 in central Japan. Antimicrob Agents Chemother 49, 137–143. doi: 10.1128/AAC.49.1.137-143.2005 15616287 PMC538884

[B27] Johnson S. R. Grad Y. Ganakammal S. R. Burroughs M. Frace M. Lipsitch M.. (2014). *In vitro* selection of neisseria gonorrhoeae mutants with elevated MIC values and increased resistance to cephalosporins. Antimicrob Agents Chemother 58, 6986–6989. doi: 10.1128/AAC.03082-14 25199775 PMC4249396

[B28] Johnson J. E. Lackner L. L. Hale C. A. de Boer P. A. J. (2004). ZipA is required for targeting of DMinC/DicB, but not DMinC/MinD, complexes to septal ring assemblies in escherichia coli. J. Bacteriol 186, 2418–2429. doi: 10.1128/JB.186.8.2418-2429.2004 15060045 PMC412171

[B29] Johnson S. R. Sandul A. L. Parekh M. Wang S. A. Knapp J. S. Trees D. L. (2003). Mutations causing *in vitro* resistance to azithromycin in neisseria gonorrhoeae. Int. J. Antimicrob Agents 21, 414–419. doi: 10.1016/S0924-8579(03)00039-6 12727073

[B30] Lewis D. A. (2015). Will targeting oropharyngeal gonorrhoea delay the further emergence of drug-resistant neisseria gonorrhoeae strains? Sexually Transmitted Infect 91, 238–240. doi: 10.1136/sextrans-2014-051730 25911525

[B31] Li H. (2011). A statistical framework for SNP calling, mutation discovery, association mapping and population genetical parameter estimation from sequencing data. Bioinformatics 27, 2987–2993. doi: 10.1093/bioinformatics/btr509 21903627 PMC3198575

[B32] Li H. Durbin R. (2009). Fast and accurate short read alignment with burrows-wheeler transform. Bioinformatics 25, 1754–1760. doi: 10.1093/bioinformatics/btp324 19451168 PMC2705234

[B33] Li H. Handsaker B. Wysoker A. Fennell T. Ruan J. Homer N.. (2009). The sequence Alignment/Map format and SAMtools. Bioinformatics 25, 2078–2079. doi: 10.1093/bioinformatics/btp352 19505943 PMC2723002

[B34] Low H. H. Moncrieffe M. C. Löwe J. (2004). The crystal structure of ZapA and its modulation of FtsZ polymerisation. J. Mol. Biol. 341, 839–852. doi: 10.1016/j.jmb.2004.05.031 15288790

[B35] Lyu M. Moseng M. A. Reimche J. L. Holley C. L. Dhulipala V. Su C.-C.. (2020). Cryo-EM structures of a gonococcal multidrug efflux pump illuminate a mechanism of drug recognition and resistance. mBio 11, 1–15. doi: 10.1128/mBio PMC7251214 32457251

[B36] Martin I. M. C. Ison C. A. Aanensen D. M. Fenton K. A. Spratt B. G. (2004). Rapid sequence-based identification of gonococcal transmission clusters in a Large metropolitan area. J. Infect. Dis. 189, 1497–1505. doi: 10.1086/383047 15073688

[B37] McArthur A. G. Waglechner N. Nizam F. Yan A. Azad M. A. Baylay A. J.. (2013). The comprehensive antibiotic resistance database. Antimicrob Agents Chemother 57, 3348–3357. doi: 10.1128/AAC.00419-13 23650175 PMC3697360

[B38] McLean C. A. Wang S. A. Hoff G. L. Dennis L. Y. Trees D. L. Knapp J. S.. (2004). The emergence of neisseria gonorrhoeae with decreased susceptibility to azithromycin in Kansas city, Missouri 1999 to 2000. Sexually Transmitted Dis. 31, 73–78. doi: 10.1097/01.OLQ.0000109514.91508.FC 14743069

[B39] Moherdaui F. Vuylsteke B. Siqueira L. F. G. dos Santos M. Q. Jr. Jardim M. L. de Brito A. M.. (1998). Validation of national algorithms for the diagnosis of sexually transmitted diseases in Brazil: results from a multicentre study. Sex Transm Inf 74, 38–43. 10023352

[B40] Murtagh F. Legendre P. (2014). Ward’s hierarchical agglomerative clustering method: Which algorithms implement ward’s criterion? J. Classification 31, 274–295. doi: 10.1007/s00357-014-9161-z

[B41] Ohneck E. A. Zalucki Y. M. Johnson P. J. T. Dhulipala V. Golparian D. Unemo M.. (2011). A novel mechanism of high-level, broad-spectrum antibiotic resistance caused by a single base pair change in neisseria gonorrhoeae. mBio 2, 1–8. doi: 10.1128/mBio.00187-11 PMC3175627 21933917

[B42] Ohnishi M. Saika T. Hoshina S. Iwasaku K. Nakayama S. I. Watanabe H.. (2011). Ceftriaxone-resistant neisseria gonorrhoeae, Japan. Emerging Infect. Dis. 17, 148–149. doi: 10.3201/eid1701.100397 PMC3204624 21192886

[B43] Olesky M. Hobbs M. Nicholas R. A. (2002). Identification and analysis of amino acid mutations in porin IB that mediate intermediate-level resistance to penicillin and tetracycline in neisseria gonorrhoeae. Antimicrob Agents Chemother 46, 2811–2820. doi: 10.1128/AAC.46.9.2811-2820.2002 12183233 PMC127413

[B44] Olesky M. Zhao S. Rosenberg R. L. Nicholas R. A. (2006). Porin-mediated antibiotic resistance in neisseria gonorrhoeae: Ion, solute, and antibiotic permeation through PIB proteins with penB mutations. J. Bacteriol 188, 2300–2308. doi: 10.1128/JB.188.7.2300-2308.2006 16547016 PMC1428387

[B45] Osaka K. Takakura T. Narukawa K. Takahata M. Endo K. Kiyota H.. (2008). Analysis of amino acid sequences of penicillin-binding protein 2 in clinical isolates of neisseria gonorrhoeae with reduced susceptibility to cefixime and ceftriaxone. J. Infect Chemother 14, 195–203. doi: 10.1007/s10156-008-0610-7 18574654

[B46] Palace S. G. Wang Y. Rubin D. H. F. Welsh M. A. Mortimer T. D.. (2020). RNA Polymerase mutations cause cephalosporin resistance in clinical neisseria gonorrhoeae isolates. eLIFE 9, 1–22. doi: 10.7554/eLife.51407 PMC7012608 32011233

[B47] Powell A. J. Tomberg J. Deacon A. M. Nicholas R. A. Davies C. (2009). Crystal structures of penicillin-binding protein 2 from penicillin-susceptible and -resistant strains of neisseria gonorrhoeae reveal an unexpectedly subtle mechanism for antibiotic resistance. J. Biol. Chem. 284, 1202–1212. doi: 10.1074/jbc.M805761200 18986991 PMC2613624

[B48] Ropp P. A. Hu M. Olesky M. Nicholas R. A. (2002). Mutations in ponA, the gene encoding penicillin-binding protein 1, and a novel locus, penC, are required for high-level chromosomally mediated penicillin resistance in neisseria gonorrhoeae. Antimicrob Agents Chemother 46, 769–777. doi: 10.1128/AAC.46.3.769-777.2002 11850260 PMC127492

[B49] Rouquette-Loughlin C. E. Reimche J. L. Balthazar J. T. Dhulipala V. Gernert K. M.. (2018). Mechanistic basis for decreased antimicrobial susceptibility in a clinical isolate of neisseria gonorrhoeae possessing a mosaic-like mtr efflux pump locus. Mol. Biol. Physiol. 9, 1–15. doi: 10.1128/mBio.02281-18 PMC6282211 30482834

[B50] Rowley J. Vander Hoorn S. Korenromp E. Low N. Unemo M. Abu-Raddad L. J.. (2019). Chlamydia, gonorrhoea, trichomoniasis and syphilis: Global prevalence and incidence estimate. Bull. World Health Organ 97, 548–562. doi: 10.2471/BLT.18.228486 31384073 PMC6653813

[B51] Seemann T. (2014). Prokka: Rapid prokaryotic genome annotation. Bioinformatics 30, 2068–2069. doi: 10.1093/bioinformatics/btu153 24642063

[B52] Shafer W. M. Folster J. P. (2006). Towards an understanding of chromosomally mediated penicillin resistance in neisseria gonorrhoeae: Evidence for a porin-efflux pump collaboration. J. Bacteriol 188, 2297–2299. doi: 10.1128/JB.188.7.2297-2299.2006 16547015 PMC1428396

[B53] Tønjum T. Caugant D. A. Dunham S. A. Koomey M. (1998). Structure and function of repetitive sequence elements associated with a highly polymorphic domain of the neisseria meningitidis PilQ protein. Mol. Microbiol. 29, 111–124. doi: 10.1046/j.1365-2958.1998.00910.x 9701807

[B54] Tomberg J. Unemo M. Ohnishi M. Davies C. Nicholas R. A. (2013). Identification of amino acids conferring high-level resistance to expanded-spectrum cephalosporins in the penA gene from neisseria gonorrhoeae strain H041. Antimicrob Agents Chemother 57, 3029–3036. doi: 10.1128/AAC.00093-13 23587946 PMC3697319

[B55] Unemo M. (2015). Current and future antimicrobial treatment of gonorrhoea - the rapidly evolving neisseria gonorrhoeae continues to challenge. BMC Infect. Dis. 15, 1–15. doi: 10.1186/s12879-015-1029-2 26293005 PMC4546108

[B56] Unemo M. Golparian D. Nicholas R. Ohnishi M. Gallay A. Sednaouie P. (2012). High-level cefixime- and ceftriaxone-resistant neisseria gonorrhoeae in France: Novel penA mosaic allele in a successful international clone causes treatment failure. Antimicrob Agents Chemother 56, 1273–1280. doi: 10.1128/AAC.05760-11 22155830 PMC3294892

[B57] Unemo M. Golparian D. Sánchez-Busó L. Grad Y. Jacobsson S. Ohnishi M.. (2016). The novel 2016 WHO neisseria gonorrhoeae reference strains for global quality assurance of laboratory investigations: Phenotypic, genetic and reference genome characterization. J. Antimicrob Chemother 71, 3096–3108. doi: 10.1093/jac/dkw288 27432602 PMC5079299

[B58] Unemo M. Golparian D. Stary A. Eigentler A. (2011). First neisseria gonorrhoeae strain with resistance to cefixime causing gonorrhoea treatment failure in Austria 2011. Euro Surveillance 16, 1–3. doi: 10.2807/ese.16.43.19998-en 22085601

[B59] Unemo M. Lahra M. M. Cole M. Galarza P. Ndowa F. Martin I.. (2019). World health organization global gonococcal antimicrobial surveillance program (WHO GASP): Review of new data and evidence to inform international collaborative actions and research efforts. Sexual Health 16, 412–425. doi: 10.1071/SH19023 31437420 PMC7035961

[B60] Unemo M. Nicholas R. A. (2012). Emergence of multidrug-resistant, extensively drug-resistant and untreatable gonorrhea. Future Microbiol. 7, 1401–1422. doi: 10.2217/fmb.12.117 23231489 PMC3629839

[B61] Unemo M. Ross J. D. C. Serwin A. B. Gomberg M. Cusini M. Jensen J. S. (2020). 2020 European guideline for the diagnosis and treatment of gonorrhoea in adults. Int. J. STD AIDS. 0, 1–7. doi: 10.1177/0956462420949126 33121366

[B62] Unemo M. Shafer W. M. (2014). Antimicrobial resistance in neisseria gonorrhoeae in the 21st century: Past, evolution, and future. Clin. Microbiol. Rev. 27, 587–613. doi: 10.1128/CMR.00010-14 24982323 PMC4135894

[B63] Vincent L. R. Kerr S. R. Tan Y. Tomberg J. Raterman E. L.. (2018). *In vivo*-selected compensatory mutations restore the fitness cost of mosaic penA alleles that confer ceftriaxone resistance in neisseria gonorrhoeae. mBio 9, 1–18. doi: 10.1128/mBio.01905-17 PMC5885032 29615507

[B64] Wadsworth C. B. Arnold B. J. Sater M. R. A. Grad Y. H. (2018). Azithromycin resistance through interspecific acquisition of an epistasis-dependent efflux pump component and transcriptional regulator in neisseria gonorrhoeae. mBio 9, 1–15. doi: 10.1128/mBio.01419-18 PMC6083905 30087172

[B65] Zarantonelli L. Borthagaray G. Lee E.-H. Shafer W. M. (1999). Decreased azithromycin susceptibility of neisseria gonorrhoeae due to mtrR mutations. Antimicrob Agents Chemother 43, 2468–2472. doi: 10.1128/AAC.43.10.2468 10508026 PMC89502

[B66] Zhao S. Duncan M. Tomberg J. Davies C. Unemo M. Nicholas R. A. (2009). Genetics of chromosomally mediated intermediate resistance to ceftriaxone and cefixime in neisseria gonorrhoeae. Antimicrob Agents Chemother 53, 3744–3751. doi: 10.1128/AAC.00304-09 19528266 PMC2737842

[B67] Zhao S. Tobiason D. M. Hu M. Seifert H. S. Nicholas R. A. (2005). The penC mutation conferring antibiotic resistance in neisseria gonorrhoeae arises from a mutation in the PilQ secretin that interferes with multimer stability. Mol. Microbiol. 57, 1238–1251. doi: 10.1111/j.1365-2958.2005.04752.x 16101998 PMC2673695

