# Supplementary material for: In vitro selection of Neisseria gonorrhoeae unveils novel mutations associated with extended-spectrum cephalosporin resistance
Source: Front Cell Infect Microbiol. 2022 Jul 27;12:924764. doi: 10.3389/fcimb.2022.924764 (PMC9363574; doi:10.3389/fcimb.2022.924764)
Supplement: Supplementary file 1 [file Table_1.docx]

Supplementary Material

Table S1. Mutations in coding genes after selection for cefixime in the isolates from this study.

| **Isolate** | ***Scaffold*** | **Position in *scaffold*** | **Gene** | **Product** | **Mutation** | **Alteration** |
| --- | --- | --- | --- | --- | --- | --- |
| **M009** | NODE_5_length_79663_cov_35.177505 | 74561 | *amiC* | N-*acetylmuramoyl*-L-*alanine amidase* AmiC | G613A | A205T |
|  | NODE_17_length_50117_cov_31.568674 | 15286 | *penA* | *peptidoglycan D,D-transpeptidase* PenA | C932T | A311V |
|  | NODE_17_length_50117_cov_31.568674 | 49719 | *pilC* | *Outer membrane protein* PilC | A353G | N118S |
|  | NODE_17_length_50117_cov_31.568674 | 49969 | *pilC* | *Outer membrane protein* PilC | A103G | S35G |
|  | NODE_48_length_9518_cov_27.120009 | 2968 | *pqiB* | *Paraquat-inducible protein* B | 498delC | L167fs |
|  | NODE_58_length_6104_cov_51.932073 | 944 | *mafA1* | *Adhesin* MafA | 840dupC | Y281fs |
| **M043** | NODE_5_length_102130_cov_50.650255 | 87011 | *rpoC* | *DNA-directed RNA polymerase subunit beta* | C3427T | P1143S |
|  | NODE_21_length_34173_cov_51.241379 | 18209 | *penA* | *peptidoglycan* D,D-*transpeptidase* PenA | C1651T | P551S |
| **M107** | NODE_3_length_95270_cov_14.046278 | 87421 | *trpE* | *Anthranilate synthase component* 1 | 370_392delGGTTACGAAACCGTCTACAATTT | G124fs |
|  | NODE_10_length_64407_cov_21.486248 | 18898 | *zapA* | *Cell division protein* ZapA | 299_300insG | E101fs |

Cont. Table S1.

| **Isolate** | ***Scaffold*** | **Position in *scaffold*** | **Gene** | **Product** | **Mutation** | **Alteration** |
| --- | --- | --- | --- | --- | --- | --- |
|  | NODE_17_length_45027_cov_19.702160 | 18704 | *mafA1* | *Adhesin* MafA | 840dupC | Y281fs |
| **M107** | NODE_21_length_40705_cov_21.469762 | 2778 | *tufA* | *Elongation factor Tuf* | C845T | T282I |
|  | NODE_42_length_11398_cov_16.050306 | 2816 | *spoT* | *Bifunctional (p)ppGpp synthase/hydrolase* SpoT | G871A | G291S |
| **M110** | NODE_8_length_75393_cov_37.360016 | 45485 | *mrcA* | *Penicillin-binding protein* 1A | C1844T | A615V |
|  | NODE_10_length_67541_cov_31.913134 | 50665 | *folP* | *Dihydropteroate synthase* | T277G | W93G |
|  | NODE_11_length_53161_cov_36.293057 | 15527 | *tufA* | *Elongation factor Tuf* | C845TT | T282I |
|  | NODE_12_length_52922_cov_28.421555 | 45509 | *rpoD* | *RNA polymerase sigma factor* RpoD | 275_286delACGATGATGCCG | D92_A95del |
|  | NODE_13_length_49612_cov_38.018147 | 32388 | *ftsZ* | *Cell division protein* FtsZ | C953T | A318V |
|  | NODE_15_length_44806_cov_36.686005 | 26318 | *mafA1* | *Adhesin* MafA | 835_836insC | Y281fs |
| **M111** | NODE_5_length_30629_cov_37.658449 | 16856 | *rseP* | *Regulator of sigma-E protease* RseP | 207_208delTA | T70fs |
|  | NODE_6_length_28549_cov_35.291042 | 12585 | *penA* | *peptidoglycan D,D-transpeptidase* PenA | C1651T | P551S |
|  | NODE_6_length_28549_cov_35.291042 | 12636 | *penA* | *peptidoglycan D,D-transpeptidase* PenA | A1600G | T534A |
|  | NODE_6_length_28549_cov_35.291042 | 12773 | *penA* | *peptidoglycan D,D-transpeptidase* PenA | C1463T | A488V |

Cont. Table S1.

| **Isolate** | ***Scaffold*** | **Position in *scaffold*** | **Gene** | **Product** | **Mutation** | **Alteration** |
| --- | --- | --- | --- | --- | --- | --- |
| **M111** | NODE_17_length_23244_cov_36.247567 | 10225 | *atpB* | ATP *synthase subunit a* | 195dupA | A66fs |
|  | NODE_17_length_23244_cov_36.247567 | 15651 | *atpD1* | ATP *synthase subunit beta 1* | C881T | S294F |
|  | NODE_48_length_16670_cov_41.178807 | 7139 | *manX* | *PTS system mannose-specific EIIAB component* | A217G | T73A |
|  | NODE_52_length_15653_cov_32.039289 | 6710 | *lptG* | *LipopoKaccharide export system permease protein* LptG | 49_50insGT | V17fs |
|  | NODE_56_length_15169_cov_25.900080 | 1536 | *rpoB* | *DNA-directed RNA polymerase subunit beta* | C1237A | R413S |
|  | NODE_56_length_15169_cov_25.900080 | 5823 | *rpoC* | *DNA-directed RNA polymerase subunit beta* | C1193A | A398D |
|  | NODE_178_length_2140_cov_27.248882 | 173 | *pilE* | *Fimbrial protein* | A364T | T122S |
|  | NODE_178_length_2140_cov_27.248882 | 178 | *pilE* | *Fimbrial protein* | A359C | E120A |
|  | NODE_178_length_2140_cov_27.248882 | 179 | *pilE* | *Fimbrial protein* | G358A | E120K |
|  | NODE_3_length_109594_cov_34.027460 | 57390 | *pilQ* | *Type IV pilus biogenesis and competence protein* PilQ | G1769A | G590D |
|  | NODE_8_length_60316_cov_36.670289 | 17293 | *rpsS* | 30S *ribosomal protein* S19 | C26T | P9L |
| **M128** | NODE_8_length_60316_cov_36.670289 | 29014 | *rpoC* | *DNA-directed RNA polymerase subunit beta* | C3437T | A1146V |
|  | NODE_18_length_34895_cov_34.381328 | 18155 | *penA* | *peptidoglycan D,D-transpeptidase* PenA | C1651T | P551S |
|  | NODE_18_length_34895_cov_34.381328 | 18169 | *penA* | *peptidoglycan D,D-transpeptidase* PenA | G1637C | G546A |

Cont. Table S1.

| **Isolate** | ***Scaffold*** | | | **Position in *scaffold*** | | **Gene** | | **Product** | | **Mutation** | | **Alteration** | |
| --- | --- | --- | --- | --- | --- | --- | --- | --- | --- | --- | --- | --- | --- |
| **M128** | | | NODE_18_length_34895_cov_34.381328 | 18290 | | *penA* | | *peptidoglycan D,D-transpeptidase* PenA | | A1516C | | N506H | |
|  |  |  | NODE_18_length_34895_cov_34.381328 | 18739 | | *penA* | | *peptidoglycan D,D-transpeptidase* PenA | | G1067A | | R356H | |
|  |  |  | NODE_18_length_34895_cov_34.381328 | 18853 | | *penA* | | *peptidoglycan D,D-transpeptidase* PenA | | C953T | | A318V | |
|  |  |  | NODE_18_length_34895_cov_34.381328 | 18874 | | *penA* | | *peptidoglycan D,D-transpeptidase* PenA | | C932T | | A311V | |
|  |  |  | NODE_18_length_34895_cov_34.381328 | 33992 | | *pilC* | | *Outer membrane protein* PilC | | 70_74delTCTTC | | S24fs | |
|  |  |  | NODE_43_length_16827_cov_26.769042 | 15121 | | *ftsK* | | *DNA translocase* FtsK | | 881_883delGCA | | S294del | |
|  |  |  | NODE_47_length_15521_cov_27.764908 | 10052 | | *pilC* | | *Outer membrane protein* PilC | | 57_58insCTTCTCTACC | | F22fs | |
| **WHO F** | | | whof_1 | 242415 | | *mltG* | | *Endolytic murein transglycosylase* | | T400C | | W134R | |
|  |  |  | whof_1 | 407443 | | *cspD* | | *Cold shock-like protein* CspD | | A37G | | K13E | |
|  |  |  | whof_1 | 1617586 | | *nsrR* | | *HTH-type transcriptional repressor* NsrR | | 187_196dupGGGCTGAGGC | | L66fs | |
|  |  |  | whof_1 | 1646413 | | *penA* | | *peptidoglycan D,D-transpeptidase* PenA | | C1651T | | P551S | |
|  |  |  | whof_1 | 1945044 | | *rpoC* | | *DNA-directed RNA polymerase subunit beta* | | C3427T | | P1143S | |
|  | | |  |  | |  | |  | |  | |  | |

Cont. Table S1.

| **Isolate** | ***Scaffold*** | **Position in *scaffold*** | **Gene** | **Product** | **Mutation** | **Alteration** |
| --- | --- | --- | --- | --- | --- | --- |
| **WHO Y** | whoy_1 | 103309 | *pilQ* | *Type IV pilus biogenesis and competence protein* PilQ | 1566_1567insG | G525fs |
|  | whoy_1 | 699170 | *smpB* | SsrA-*binding protein* | 147dupA | D50fs |
|  | whoy_1 | 1161488 |  | *putative protein* | 181delA | T63fs |
|  | whoy_1 | 1371130 | *mtrD* | *Multidrug resistance protein* MexB | T1709A | M570K |
|  | whoy_1 | 1374022 | *mtrC* | *Multidrug resistance protein* MexA | T67C | S23P |
|  | whoy_1 | 1510963 | *tbp1* | *Transferrin-binding protein* 1 | 529_530insC | A178fs |
|  | whoy_1 | 1928889 | *resA* | *Thiol-disulfide oxidoreductase* ResA | 126dupC | V43fs |
|  | whoy_1 | 1948729 | *pilT* | *Twitching mobility protein* | G104A | R35Q |
|  | whoy_1 | 2013759 | *rpoB* | *DNA-directed RNA polymerase subunit beta* | A1063G | T355A |
|  | whoy_1 | 2047432 | *porB* | *Major outer membrane protein* PorB1b | C77T | A26V |

Legend**:** COG, Clusters of Orthologous Groups; nt, nucleotide; aa, amino acid; fs, frameshift.
